# Color Doppler Indices of Orbital Arterial Flow in End-Stage Renal Disease Patients; Are the Changes Related to Chronic Hemodialysis or Chronic Renal Failure?

**DOI:** 10.5812/iranjradiol.6730

**Published:** 2012-03-25

**Authors:** Hadi Rokni Yazdi, Safoura Faraji, Farokhlegha Ahmadi, Reza Shahmirzae

**Affiliations:** 1 Department of Radiology, Advanced Diagnostic and Interventional Radiology Research Center (ADIR), Imam Khomeini Hospital, Tehran University of Medical Sciences (TUMS), Tehran, Iran; 2 Department of Radiology, Imam Khomeini Hospital, Tehran University of Medical Sciences (TUMS), Tehran, Iran; 3 Department of Nephrology, Nephrology Research Center (NRC), Imam Khomeini Hospital, Tehran University of Medical Sciences (TUMS), Tehran, Iran; 4 Department of Cardiology, Tehran University of Medical Sciences (TUMS), Tehran, Iran

**Keywords:** Ultrasonography, Doppler, Color, Kidney Failure, Chronic, Renal Dialysis

## Abstract

**Background:**

Endothelial injury is a well-known complication in chronic kidney disease (CKD) and hemodialysis. One of the sites in which early vascular changes may be detected is the retina. Of course, these flow changes may not be detected in ophthalmologic exams, but it seems that color Doppler sonography of retinal arteries may be helpful in these cases.

**Objectives:**

In previous studies on CKD patients who underwent chronic hemodialysis,hemodynamic changes were noted in retinal arteries, but no study has been performed to determine which of the two processes (CKD or chronic hemodialysis) can produce these changes. In this study, we tried to answer this question.

**Patients and Methods:**

Doppler ultrasonography of the orbital vasculature including the ophthalmic artery and the central retinal artery was carried out in 17 patients (34 eyes) with chronic renal failure (CRF) who underwent hemodialysis, 17 patients (34 eyes)with CRF without a history of hemodialysis and 17 normal patients (34 eyes). The peak systolic velocity (PSV), end diastolic velocity (EDV) and resistance index were measured excluding hypertensive, diabetic patients and patients with cardiovascular disease.

**Results:**

The mean PSV and EDV were lower only in the ophthalmic artery of CRF patients irrespective of the history of hemodialysis (PSV was 35.2 in hemodialysis, 38.8 in CRF and 51.6 in normal patients, P value = 0.001 and EDV was 7.4, 9.4, 11.8, respectively, P value =0.001) with no significant difference in the resistance index of the ophthalmic artery and other parameters [EDV, PSV, Resistance Index (RI)] in the central retinal artery.

**Conclusions:**

The mean PSV and DSV in the ophthalmic artery were lower only in the ophthalmic artery of CRF patients regardless of the history of hemodialysis. No significant difference in the resistance index of the ophthalmic artery and other parameters (EDV, PSV) of the central retinal artery were noted between different groups.

These findings suggest that microvascular disease and endothelial cell dysfunction of the orbital vasculature are related to CRF and not to chronic hemodialysis.

## 1. Background

Chronic renal failure (CRF) is one of the important risk factors for cardiovascular disease due to accelerated atherosclerosis in the vascular –including the macrovascular and microvascular–bed [1].

Abnormalities of coronary and cerebral circulation in patients with end-stage renal disease have been well evaluated [1][2]. However, the data available on the effect of chronic renal disease or chronic hemodialysis on retrobulbar arterial hemodynamic changes are limited.

Doppler ultrasound is a non-expensive, non-invasive method for quantitative evaluation of orbital vessel pathologies. It was first introduced by Erickson et al. [3] in 1989 and has been frequently used in the diagnosis of different ocular diseases since then.

There are only few studies on the evaluation of retrobulbar blood hemodynamics by color Doppler ultrasonography in hemodialysis and CRF patients.

Tosun et al. [4] evaluated the effect of a single hemodialysis session on retrobulbar blood hemodynamics by color Doppler ultrasonography in 35 patients before and after dialysis and found out a reduction in systolic and diastolic blood flow in ophthalmic, central retinal and posterior cilliary arteries after the hemodialysis session.

Saygili et al. [5] evaluated Doppler indices in central retinal and posterior cilliary arteries in 20 patients and compared them with 22 controls and found significantly reduced resistance index (RI) values and increased blood flow velocities in both arteries in end stage renal disease (ESRD) patients undergoing hemodialysis compared to the control group, but they did not evaluate ESRD patients who were not on hemodialysis, so they noted that the changes in blood flow may be either the result of ESRD or hemodialysis treatment so they suggested further studies.

## 2. Objectives

In this study we tried to determine which of the two processes; chronic kidney disease (CKD) or chronic hemodialysis can be associated with these changes.

We considered three groups of patients; normal kidney function, patients with CRF and patients with CRF that had undergone dialysis and then compared Doppler indices–end diastolic velocity (EDV), peak systolic velocity (PSV) and RI–in retinal arteries in these three groups. In this study we intended to find out the association of hemodynamic changes in ophthalmic arteries–evaluated by color Doppler–with CRF and dialysis.

## 3. Patients and Methods

This study was performed from August 2008 to February 2010. Three groups were considered. Group A consisted of 17 ESRD patients undergoing hemodialysis (11 male, six female) (age range, 17-45 years) for 10 to 180 months (3 days in a week and four hours in each cession using poly sulfan filter and bicarbonate buffer system). Group B consisted of 17 patients with ESRD with no need of hemodialysis (nine male, eight female) (age range, 23-48 years) and finally; group C consisted of 17 healthy patients selected from patients referred for unrelated problems to an orthopedic clinic. Group C patients (nine male, eight female) (age range, 22-45) had no history of hypertention or diabetes and had a normal renal function (control group). All subjects of the three groups were selected by simple sequential sampling.

Exclusion criteria were history of diabetes and hypertension, cardiovascular disorders, age greater than 50 years and any ophthalmic disorder that may affect the Doppler study (history of trauma, glaucoma and retinal detachment). The above mentioned conditions were the most common reasons that could affect sonographic and Doppler measurements of the study.

The patients and controls had no symptom of ocular diseases, but no general ophthalmological exam was performed for them.

**Figure 1:**
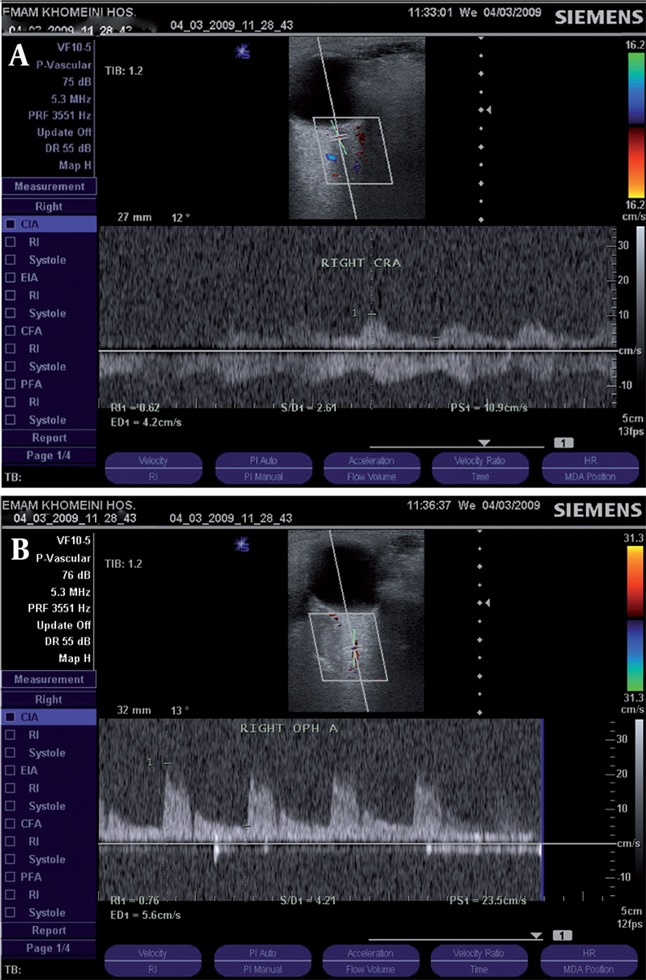
A 23-year-old female with PCKD and a history of 5-month dialysis. A, Doppler spectrum of the central retinal artery (CRA); B, Ophthalmic artery (OA)

Each patient underwent Doppler ultrasound of both ophthalmic arteries (OAs) and the central retinal artery (CRA) using a 5.3 MHz multifrequency linear array probe (SONOLINE G40TM, Siemens Medical Solutions USA, Inc., Malvern, Pennsylvania, United States) (Figure 1). The patients lay in a supine position during the examination, acoustic gel was applied on shut eyelids and the probe was placed without applying any pressure to the globe. To minimize the exertion of pressure on the globe, the examiner supported his hand on the subject’s forehead. The same radiologist performed all Doppler examinations. The radiologist was unaware of the patient’s diagnosis. In CRA, Doppler measurements were taken 1 cm distal to the optic nerve in the ophthalmic artery when it crosses the optic nerve. When the desired measurement site was identified by color Doppler, the position of the transducer was adjusted to afford an angle of less than 60. The sample volume was adjusted as minimum as possible (1 mm) to include the entire inner diameter of the vessels. Pulse repetition frequency (PRF) was 4340 Hz for the ophthalmic artery and 2441 Hz for the CRA. PSV (cm/s), EDV (cm/s) and RI (RI = PSV-EDV/PSV) were determined from spectral waveform.

The demographic data, CRF etiology, weekly hemodialysis time and the beginning time of hemodialysis were reported for each patient.

Finally, all the data were analyzed using SPSS 11.5 software, and one way ANOVA and a P value less than 0.05 was considered significant. The normality of data was assessed by Kolmogorov-Smirnov test. Pairwise comparisons were done by Tukey test.

This study was approved by the ethical committee of Tehran University of Medical Sciences.

## 4. Results

The means age of patients was 32.3 years for group A, 36.2 years for group B and 31.4 years for group C. The mean age and gender distribution were not statistically different between the three groups (both P values > 0.05).

The most common etiology of CRF in group A was unknown probably because of CRF and a glomerular filtration rate (GFR) of less than 15 in those patients which obviated biopsy and pathological study, and in group B the most common etiology of CRF was glomerulonephritis (Table 1).

**Table 1:** Etiology of CKD in Two A (Hemodialysis) and B (CKD) Groups

	**Group**		**Total, n = 34**
	**Hemodialysis, n = 17**	**CKD a, n = 17**	
Unknown, No.	8	3	11
PCKD **a**, No.	2	3	5
GN **a**, No.	3	9	12
Reflux, No.	2	1	3
IN **a**, No.	2	1	3

^a^ Abbreviations: CKD, chronic kidney disease; GN, glomerulonephritis; IN, interstitial nephritis; PCKD, polycystic kidney disease

The results of Doppler parameters in three groups are summarized in Table 2. Comparison of the mean of Doppler parameters of CRA in the three groups showed no significant difference in PSV, EDV and RI indices between normal patients and the two other groups (P value = 0.57, 0.31, 0.64, respectively for PSV, EDV and RI).

**Table 2:** Mean of Doppler Parameters in Groups A (Hemodialysis), B (CKD) and C (Control)

	**Mean**	**Minimum**	**Maximum**	***P *value**
RPSVCRA a, cm/s				
hemodialysis	11.06	4.60	17.70	0.572
CKD	13.22	3.20	39.90	
normal	11.40	9.00	14.10	
total	12.02	3.20	39.90	
REDVCRA a, cm/s				
hemodialysis	4.49	0.00	8.20	0.317
CKD	6.46	2.80	24.00	
normal	3.96	3.00	5.10	
total	5.20	0.00	24.00	
RRICRA a, cm/s				
hemodialysis	0.63	0.54	1.00	0.644
CKD	0.60	0.48	0.77	
normal	0.62	0.52	0.68	
total	0.61	0.48	1.00	
LPSVCRA a, cm/s				
hemodialysis	11.88	5.30	20.60	0.194
CKD	10.99	7.90	18.30	
normal	13.36	10.60	16.90	
total	11.80	5.30	20.60	
LEDVCRA a, cm/s				
hemodialysis	3.99	0.00	5.60	0.126
CKD	4.14	3.20	7.10	
normal	5.00	3.20	6.10	
total	4.25	0.00	7.10	
LRICRA a, cm/s				
hemodialysis	0.67	0.54	1.00	0.155
CKD	0.61	0.53	0.68	
normal	0.64	0.52	0.74	
total	0.64	0.52	1.00	
RPSVOA a, cm/s				
hemodialysis	35.26	20.00	44.70	< 0.0001
CKD	38.88	23.60	59.10	
normal	51.69	39.10	68.10	
total	39.97	20.00	68.10	
REDVOA a, cm/s				
hemodialysis	7.42	3.50	11.50	
CKD	9.44	4.70	13.10	0.001
normal	11.80	8.90	17.10	
total	9.11	3.50	17.10	
RRIOA a, cm/s				
hemodialysis	0.78	0.65	0.91	0.164
CKD	0.73	0.58	0.88	
normal	0.75	0.69	0.80	
total	0.75	0.58	0.91	
LPSVOA a, cm/s				
hemodialysis	39.09	23.60	64.20	0.012
CKD	39.32	25.10	51.50	
normal	52.35	36.40	72.30	
total	41.77	23.60	72.30	
LEDVOA a, cm/s				
hemodialysis	8.37	4.80	13.60	0.002
CKD	10.22	3.30	13.50	
normal	13.41	8.40	19.50	
total	10.12	3.30	19.50	

^a^ Abbreviations: LEDVCRA, left end diastolic velocity in central retinal artery; LEDVOA, left end diastolic velocity in ophthalmic artery; LPSVCRA, left peak systolic velocity in central retinal artery; lPSVOA, Left peak systolic velocity in ophthalmic artery; LRICRA, left resistance index in central retinal artery;RRICRA, right resistance index in central retinal artery; RRIOA, right resistance index in ophthalmic artery; RPSVCRA, right peak systolic velocity in central retinal artery; RPSVOA, right peak systolic velocity in ophthalmic artery; REDVCRA, right end diastolic velocity in central retinal artery; REDVOA, right end diastolic velocity in ophthalmic artery

But comparison of the mean of Doppler parameters of OA in the three groups showed significant difference in PSV and EDV indices of both eyes, between the normal patients and the two other groups (P value = 0.001). These parameters were lower in group A and group B compared to group C (Table 2). And finally no difference was seen between RI in these three groups, neither for the CRA, nor for the OA.

## 5. Discussion

ESRD has many complications, such as metabolic, endocrine, macrovascular and microvascular complications. Ocular complications include choroidal and retinal vasculopathy, optic neuropathy and cataract [6]. Some of these findings are due to hypertension or diabetes, but in this study we enrolled ESRD patients who did not have diabetes or hypertension and we evaluated ocular Doppler parameters.

EDV and PSV for OA in both groups of A and B were significantly lower than normal patients (group C) in each eye. But other parameters, including RI was not significantly different between the three groups.

In the CRA, there was no difference between Doppler parameters in the three groups including PSV, EDV and RI. Our findings for CRA were different from findings of Saygili et al.’s study [5] which evaluated Doppler indices in the central retinal and the posterior cilliary arteries of 20 patients and 22 controls and found significantly reduced RI values and increased blood flow velocities in both arteries in ESRD patients undergoing hemodialysis, compared to the control group. They noted that their findings may be related to either increased flow or vasoconstriction of proximal vessels, but increased velocities in the OA in our patients is similar to their findings in the cilliary arteries which may be due to the same reason that they noted.

These findings suggest that only CRF is associated with Doppler indices in the OA and hemodialysis does not have any association with ophthalmic hemodynamics.
